# Peripheral Blood B-Lymphocytes Are Involved in Lymphocystis Disease Virus Infection in Flounder (*Paralichthys olivaceus*) via Cellular Receptor-Mediated Mechanism

**DOI:** 10.3390/ijms23169225

**Published:** 2022-08-17

**Authors:** Xiuzhen Sheng, Jing Zeng, Ying Zhong, Xiaoqian Tang, Jing Xing, Heng Chi, Wenbin Zhan

**Affiliations:** 1 Laboratory of Pathology and Immunology of Aquatic Animals, KLMME, Ocean University of China, 5 Yushan Road, Qingdao 266003, China; 2 Function Laboratory for Marine Fisheries Science and Food Production Processes, Qingdao National Laboratory for Marine Science and Technology, Qingdao 266071, China

**Keywords:** B-lymphocytes, lymphocystis disease virus, 27.8 kDa-receptor protein, infection, replication, flounder (*Paralichthys olivaceus*)

## Abstract

Previous studies imply that peripheral blood leukocytes (PBLs) may play an important role in systemic lymphocystis disease virus (LCDV) dissemination, but whether the PBLs are susceptible and permissive to LCDV infection and the dissemination mechanism need to be clarified. In this study, LCDV was firstly confirmed to infect the PBLs in flounder (*Paralichthys olivaceus*) in vivo, and to replicate in PBLs in vitro. Subsequently, the 27.8 kDa receptor protein (27.8R), a functional receptor mediating LCDV infection in flounder gill cells, was shown to locate on the cell membrane of PBLs and co-localize with LCDV in PBLs, while blocking of the 27.8R via pre-incubation of anti-27.8R MAb with the PBLs could obviously inhibit LCDV infection, revealing the 27.8R as a receptor for LCDV entry into PBLs. Multicolor fluorescence imaging studies verified that IgM^+^ and IgD^+^ B-lymphocyte were involved in LCDV infection. In the sorted IgM^+^ B-cells, 27.8R^+^ and LCDV^+^ signals were simultaneously observed, and LCDV copy numbers increased with time, indicating that IgM^+^ B-cells expressed the 27.8R and were permissive to LCDV infection. Furthermore, the dynamic changes of IgM^+^, 27.8R^+^, LCDV^+^ and LCDV^+^/IgM^+^ PBLs were monitored during the early phase of LCDV infection. It was found that the percentage of IgM^+^ B-cells in PBLs clearly declined first and then increased, suggesting LCDV infection facilitated damage to B-cells, whereas the amounts of 27.8R^+^ and LCDV^+^ PBLs, as well as LCDV-infected IgM^+^ B-cells, showed an opposite trend. These results proved that IgM^+^ B-lymphocytes could be infected by LCDV via a receptor-mediated mechanism and support viral replication, which provided novel insights for the first time into the role of B-lymphocytes in LCDV dissemination and pathogenesis in teleost fish.

## 1. Introduction

Lymphocystis disease virus (LCDV), a member of the genus *lymphocystivirus* within the *Iridoviridae* family [1,2], is the etiological agent of lymphocystis disease. Lymphocystis disease is a well-known disease that is characterized by the presence of pearl-like nodules on the skin, fins and internal organs of the fish [3], which has affected more than 140 marine, brackish and freshwater fish worldwide [4], and new LCDV isolations are still continuously found in wild and cultured fish species [5,6,7]. The incidence rate of this disease may reach 70%, causing significant economic losses for the aquaculture industry [8]. Although lymphocystis disease is a self-limiting and rarely fatal disease, and the lesions generally can resolve and fish may recover after several weeks [9], fish with the typical symptoms appear disfigured and are thus less saleable. Commonly, the affected fish may become more susceptible to secondary infection by other pathogens which results in high mortality [2]. To shed light on LCDV infection mechanisms and develop effective prophylactic measures, considerable attention is focused on virus-host interaction and transmission route of LCDV [10,11,12,13,14,15,16,17]. Remarkably, a 27.8 kDa membrane protein from flounder (*Paralichthys olivaceus*) gill (FG) cells is identified as a cellular receptor to mediate LCDV binding and infection through interaction with a 32 kDa viral attachment protein of LCDV [12,14,18,19], and the 27.8 kDa receptor (27.8R) protein is found to be widely distributed in the tissues of flounder [20] and turbot (*Scophthalmus maximus*), even in some peripheral blood leukocytes (PBLs) in turbot [21]. Moreover, a 37.6 kDa membrane protein is also found to be involved in binding and infection to LCDV in FG cells [16], but its role in LCDV invasion of host cells is still unclear. Recently, the 27.8R have been further identified as voltage dependent anion channel protein 2 (VDAC2) and receptor of activated protein C kinase 1 (RACK1) of flounder (*P. olivaceus*) [22], and they facilitate LCDV entry into FG cells via a caveolae-mediated endocytosis mechanism [15]. These novel findings will contribute to analyzing the tissue tropism and dissemination route of LCDV in vivo. LCDV is either horizontally transmitted via direct contact and external trauma [3], or vertically transmitted to the fertilized eggs [13]. However, the dissemination mechanism of LCDV between different tissues after viral entry into fish is largely unknown.

Knowledge of cell types involved in systemic virus dissemination is fundamental for the understanding of LCDV pathogenesis. A study using histopathological examination in tissues of red drum (*Sciaenops ocellatus*) suffering from lymphocystis disease, points to a systemic infection involving a variety of internal organs probably via LCDV entry into the bloodstream [23]. Moreover, LCDV has been detected in in vitro cultured leukocytes from the head kidney of gilthead seabream (*Sparus aurata*) [24], and the LCDV genome was also examined in blood samples of the asymptomatic gilthead seabream broodstock in a farm with previous reports of lymphocystis disease [13]. Our previous studies indicate that LCDV can be widely detected in all tissues of flounder and turbot after intramuscular injection with LCDV [20,21], as well as in some PBLs but not in red blood cells of turbot, and LCDV copy numbers increase with the time in various tissues [21], implying that PBLs may play an important role in LCDV dissemination between different tissues leading to a systemic infection. Nonetheless, whether the PBLs are susceptible and permissive to LCDV infection and which subsets of leukocytes are involved needs further clarification.

In this study, LCDV in the PBLs was firstly detected by indirect immunofluorescence assay (IFA), flow cytometry and quantitative real time PCR (qPCR) after in vivo infection of flounder. The viral replication in the PBLs post in vitro LCDV infection was then confirmed by qPCR and transmission electron microscopy (TEM). Subsequently, IFA and immunogold electron microscopy (IEM) were carried out to clarify whether the 27.8R involved in LCDV infection of PBLs, and antibody blocking assay further demonstrated the function of the 27.8R as a receptor for LCDV infection of PBLs. On this basis, we identified the subset of leukocytes which was susceptible to LCDV infection, while the dynamic changes of IgM^+^, the 27.8R^+^, LCDV^+^ and LCDV-infected IgM^+^ PBLs were monitored at the early phase of infection. Our results show that IgM^+^ B-lymphocytes are susceptible to direct infection by LCDV through the 27.8R and support viral replication, which will provide novel insights into the role of B-lymphocytes in LCDV dissemination and pathogenesis in fish.

## 2. Results

### 2.1. LCDV Infects the PBLs in Flounder In Vivo

**Figure 1:**
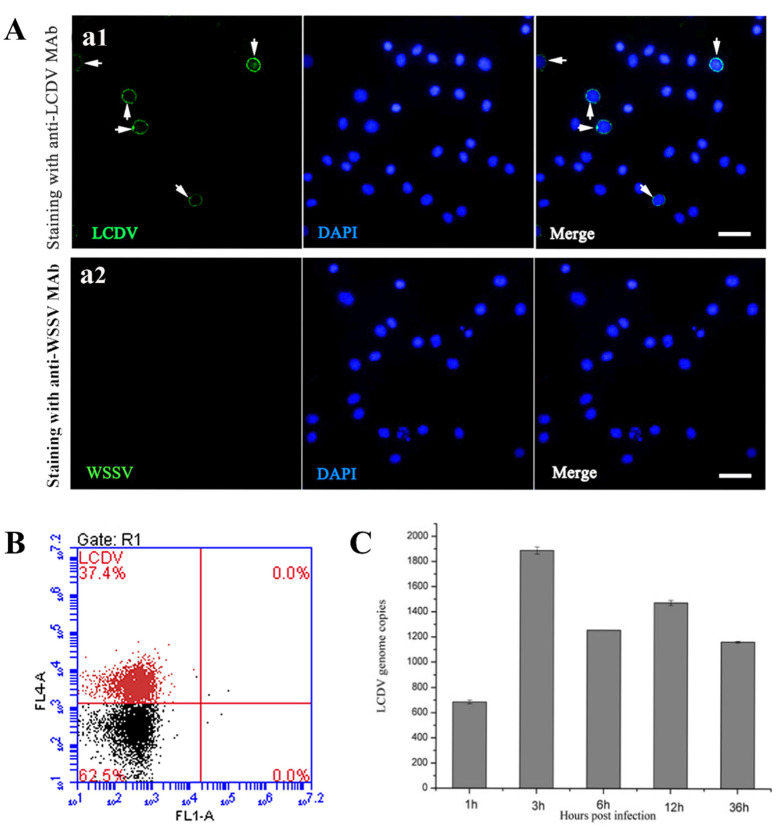
Detection of LCDV in peripheral blood leukocytes (PBLs) of LCDV-infected flounder in vivo. (**A**) Anti-LCDV MAb staining of the isolated PBLs, showing positive green signals in some PBLs (white arrows) (**a1**); Anti-WSSV MAb staining of PBLs presented no green signals as negative control (**a2**). (**B**) Flow cytometry analysis indicated the percentage of LCDV^+^ cells in PBLs by scatter diagram. (**C**) The LCDV copy numbers in PBLs of LCDV-infected flounder at different time points. Cell nuclei were counterstained in blue by DAPI. Scale bar = 10 μm. Error bars represent standard deviations (SD, *n* = 3).

To ascertain whether the PBLs were involved in LCDV infection, the IFA was performed in isolated PBLs of in vivo LCDV-infected flounder. The results indicated that LCDV-positive (LCDV^+^) green signals were present in a small portion of PBLs that contained a large, central nucleus and small amount of cytoplasm (Figure 1(a1)), but no positive fluorescence was seen in the negative control stained with anti-white spot syndrome virus (WSSV) monoclonal antibody (MAb) (Figure 1(a2)). Cell nuclei of the PBLs were counterstained in blue by DAPI. As shown by flow cytometry analysis, the percentage of LCDV^+^ cells accounted for about 37% of PBLs (Figure 1B). qPCR results demonstrated that the copy numbers of LCDV in PBLs from the infected-flounder at different time points showed a trend of increase first and then decrease, reaching a higher peak value (1.92 × 10^3^ copies) at 3 h post infection (hpi), and a second slightly lower peak value (1.48 × 10^3^ copies) at 12 hpi (Figure 1C). These results revealed that LCDV could infect PBLs in flounder in vivo.

### 2.2. LCDV Replicates in PBLs In Vitro

**Figure 2:**
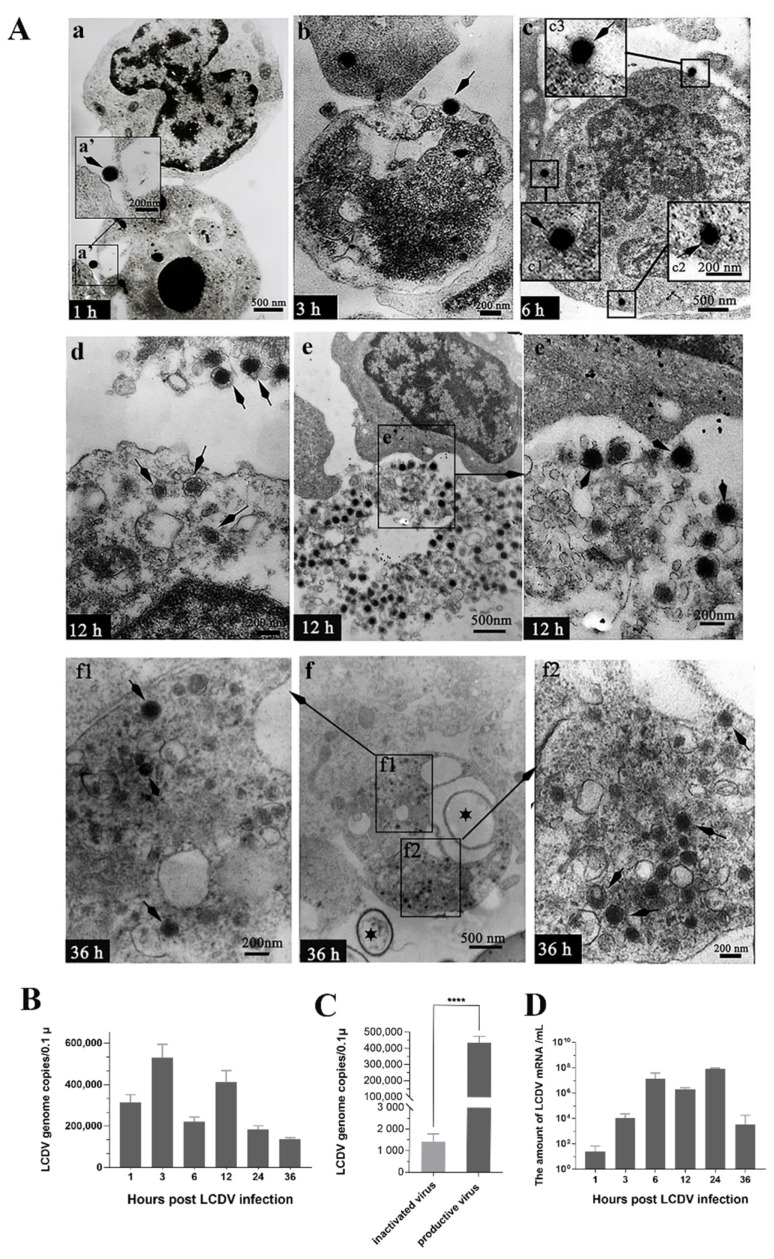
LCDV replication in flounder PBLs post in-vitro LCDV infection at different time points. (**A**) TEM micrographs of LCDV replication. The arrows showing LCDV particles in infected cells at 1 h, 3 h, 6 h, 12 h and 36 h post infection in vitro, respectively (**a**–**f**). (**a’**) Higher magnification view of insert area from (**a**); (**c1**–**c3**) Higher magnification view of insert area from (**c**), showing LCDV particles on the cell membrane and in the cytoplasm of PBLs; (**e’**) Higher magnification view of insert area from (**e**); (**f1**,**f2**) Higher magnification view of insert area from (**f**); The star in (**f**) showing the apoptotic bodies; Scale bar = 200 nm. (**B**) The LCDV copy numbers in PBLs at different time points post in vitro LCDV infection. (**C**) The LCDV copy numbers in PBLs incubated with productive LCDV and UV-inactivated LCDV were detected at 12 hpi. **** *p* < 0.0001, unpaired *t* test. (**D**) Viral genome transcription in PBLs at different time points post LCDV infection. Error bars represent SD (*n* = 3).

To verify LCDV replication in PBLs, the ultrathin sections of PBLs at 1, 3, 6, 12 and 36 hpi were prepared and observed under TEM. The result indicated that LCDV particles showed a hexagonal outline with a dense central core and a size of about 200 nm. At 1 hpi, LCDV particles were observed to attach to the cell membrane of PBLs, and the karyopyknosis of the PBLs could be seen Figure 2(Aa). At 3 hpi and 6 hpi, LCDV particles were present in the cytoplasm and also on the surface of PBLs (Figure 2(Ab,Ac)). At 12 hpi, the viral particles and swelling of mitochondria were observed in the cytoplasm, and virus particles were even released outside the cells (Figure 2(Ad)). More notably, a karyolitic cell was found to be full of LCDV particles, but its cell membrane was dissolved and the nucleus disappeared (Figure 2(Ae)), suggesting the virus replication might cause cell disruption. At 36 hpi, virus particles at different stages of development in the sites of virus synthesis, and virions with hexagonal-shaped structures were clearly observed in the cytoplasm of PBLs, while cell blebbing was also exhibited and some apoptotic bodies were scattered around the cells (Figure 2(Af,Af1,Af2)). These results indicated LCDV could replicate in PBLs which might result in cell death and apoptosis in some degree.

To further elucidate how LCDV could actively replicate in the PBLs, the isolated PBLs from the healthy flounder were inoculated with LCDV in vitro, and the qPCR result indicated that LCDV copy numbers in PBLs from 1 hpi to 36 hpi first increased and then decreased, and reached a higher peak at 3 hpi (5.81 × 10^5^ copies) and a slightly lower peak at 12 hpi (4.33 × 10^5^ copies) (Figure 2B), showing a similar trend to that in PBLs in in-vivo-infected flounder, but with a number of higher viral copies. To eliminate the influence of the antigen phagocytosis by PBLs, the PBLs was inoculated with UV-inactivated LCDV as a control. The result demonstrated that the viral copy numbers in PBLs incubated with productive LCDV (4.81 × 10^5^ copies) were significantly higher than in those incubated with UV-inactivated LCDV (1.12 × 10^3^ copies) at 12 hpi (*p* < 0.0001) (Figure 2C). Meanwhile, the amount of LCDV mRNA ascended and peaked at 24 hpi (1.58 × 10^7^ copies) (Figure 2D). All these results indicated that LCDV could replicate in PBLs.

### 2.3. LCDV Infects the PBLs Expressing the 27.8R

**Figure 3:**
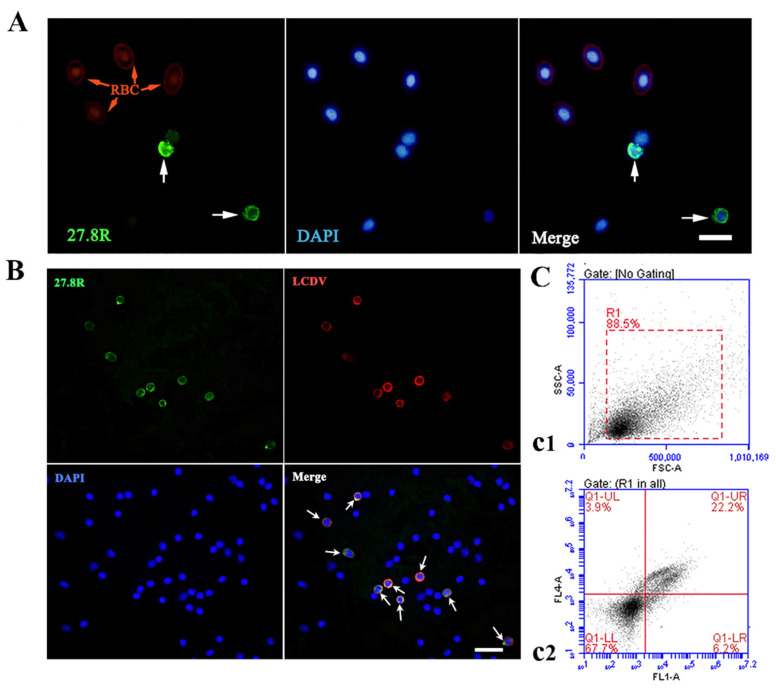
Detection of the 27.8R in whole blood cells and its co-localization with LCDV in PBLs at 3 h post in-vivo infection in flounder. (**A**) Detection of the 27.8R in whole blood cells. Red blood cells (RBCs, red arrow) were non-specifically fluoresced in red by Evan’s blue dye. The positive green signals (white arrows) represented the location of the 27.8R protein in some leukocytes. Cell nuclei were counterstained in blue by DAPI. Scale bar = 10 μm. (**B**) PBLs slide was stained with the mixtures of mouse anti-27.8R MAbs and rabbit anti-LCDV polyclonal antibody. The merged signals indicated that some PBLs (white arrows) exhibited LCDV^+^ (red fluorescence) and 27.8R^+^ (green fluorescence) double positive. Cell nuclei were counterstained in blue by DAPI. Scale bar = 20 μm. (**C**) Flow cytometry analysis showed the PBLs that gated on a FS/SS dot plot (**c1**), and the 27.8R^+^/LCDV^+^ PBLs (**c2**). Q1-UL represented single fluorescence of 27.8R^+^ cells, Q1-LR represented single fluorescence of LCDV^+^ cells, Q1-UR represented double fluorescence of 27.8R^+^/LCDV^+^ PBLs.

The whole blood cells of LCDV-infected flounder were stained using anti-27.8R MAbs, and the positive green fluorescence for the 27.8R were observed on the surface of some leukocytes, but not on red blood cells which were non-specifically fluoresced in red by Evan’s blue dye (Figure 3A). To further confirm the relationship between LCDV-infected and 27.8R-expressing leukocytes, the LCDV and 27.8R were simultaneously detected on the same slide of the isolated PBLs at 3 hpi by IFA. The results indicated that the 27.8R^+^ green fluorescence was co-localized with LCDV^+^ red fluorescence on the same PBLs (Figure 3B), revealing that LCDV infected the PBLs expressing the 27.8R. Cell nuclei were counterstained in blue by DAPI. Furthermore, about 22% of PBLs were detected as LCDV^+^/27.8R^+^ double positive by flow cytometry analysis at 3 hpi (Figure 3(c1,c2)).

### 2.4. Anti-27.8R MAb Blocks LCDV Infection of PBLs

**Figure 4:**
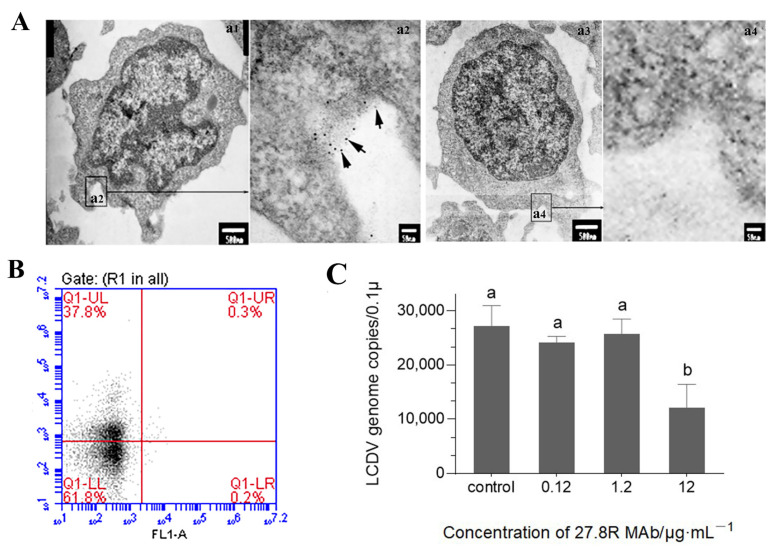
Location of the 27.8R on PBLs and antibody blocking assay using anti-27.8R MAb to inhibit LCDV infection of PBLs. (**A**) Immunogold electron micrograph of the 27.8R-expressing PBLs in healthy flounder. (**a1**,**a3**) The PBLs incubated with anti-27.8R and anti-WSSV MAb, respectively. Scale bar = 500 nm. (**a2**,**a4**) Higher magnification view of insert area from (**a1**) and (**a3**), respectively. The arrows showed gold particles representing the 27.8R. Scale bar = 50 nm. (**B**) Flow cytometry analysis showed the 27.8R^+^ cells in PBLs by scatter diagram, Q1-UL represented 27.8R^+^ cells. (**C**) The LCDV copy numbers in PBLs after pre-incubation with different concentration of anti-27.8R MAb. The different lowercase (b) indicated the significant difference between experimental groups and the control (*p* < 0.05), while (a) indicated no significant difference (*p >* 0.05). Error bars represent SD (*n* = 3).

For locating the 27.8R on PBLs, the PBLs isolated from LCDV-free flounder were observed after immunogold staining with anti-27.8R MAb 2G11 as probe by IEM. The results showed that the 27.8R labelled with gold particles was mainly located on the cell membrane, and the 27.8R-expressing PBL was characterized by a large nucleus, a thin rim of cytoplasm with small protrusions on the cell surface, representing the morphological features of lymphocytes (Figure 4(a1,a2)). In the negative control using anti-WSSV MAb, no gold particles were observed on the PBLs (Figure 4(a3,a4)). Flow cytometry analysis showed that 37.8% of PBLs were stained 27.8R-positive (Figure 4B).

To confirm if the 27.8R acted as the viral receptor for LCDV entry and infection in the PBLs, the antibody blocking assay was conducted in PBLs in vitro. The qPCR result indicated that LCDV copy numbers slightly decreased in presence of 0.12 μg/mL and 1.2 μg/mL of anti-27.8R MAb (*p* > 0.05), and significantly decreased in the presence of 12 μg/mL of anti-27.8R MAb (*p* < 0.05) as compared with the control (Figure 4C), revealing that blocking the 27.8R on the PBLs could abrogate LCDV infection.

### 2.5. IgM^+^ and IgD^+^ B-Lymphocyte Subsets Involved in LCDV Infection

**Figure 5:**
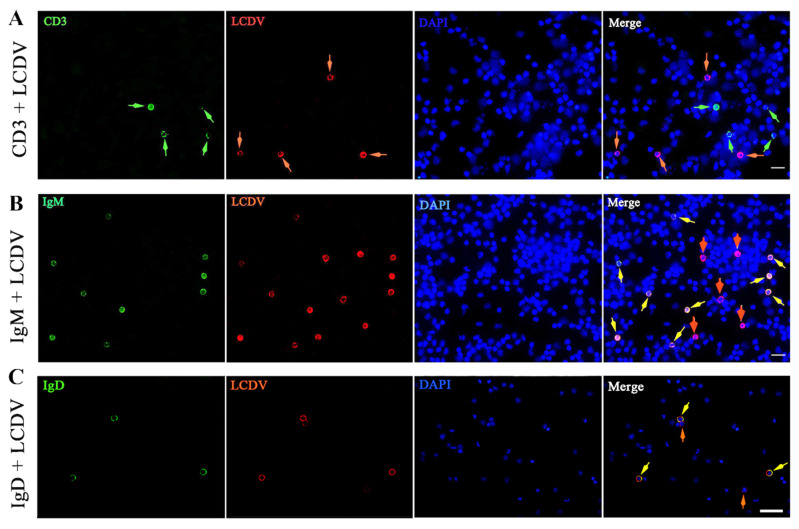
Co-fluorescent microscopy of LCDV^+^ cells and CD3^+^, IgM^+^, IgD^+^ cells in PBLs from flounder at 3 h post LCDV infection. (**A**) Rabbit anti-LCDV polyclonal antibody was paired with mouse anti-CD3ε polyclonal antibody, green and red arrows indicated CD3^+^ T-lymphocyte and LCDV^+^ PBLs, respectively. (**B**) Rabbit anti-LCDV polyclonal antibody was paired with mouse anti-IgM MAb, yellow and red arrows indicated LCDV^+^/IgM^+^ B-lymphocytes and LCDV^+^/IgM^−^ PBLs, respectively. (**C**) Rabbit anti-LCDV polyclonal antibody was paired with mouse anti-IgD MAb, yellow and red arrows indicated LCDV^+^/IgD^+^ B-lymphocytes and LCDV^+^/IgD^−^ PBLs, respectively. Cell nuclei were counterstained in blue by DAPI. Scale bar = 20 μm.

To investigate the specific subpopulation that was susceptible to LCDV infection in PBLs, co-staining of LCDV and CD3ε, IgM, IgD were performed on the same PBLs smears, respectively. The IFA results demonstrated that LCDV^+^ and CD3ε^+^ fluorescence signals were detected in different PBLs, suggesting LCDV could not infect T-lymphocytes recognized by anti-CD3ε antibody (Figure 5A). Nevertheless, LCDV^+^/IgM^+^ and LCDV^+^/IgM^−^ PBLs were observed (Figure 5B), revealing that LCDV could infect IgM^+^ B-lymphocytes and some IgM^−^ PBLs. Similarly, LCDV^+^/IgD^+^ and LCDV^+^/IgD^−^ PBLs were exhibited, indicating that LCDV could infect IgD^+^ B-lymphocytes and some IgD^−^ PBLs (Figure 5C). Cell nuclei were counterstained in blue by DAPI.

### 2.6. Co-Localization of LCDV and the 27.8R on the Sorted IgM^+^ B-Lymphocytes

**Figure 6:**
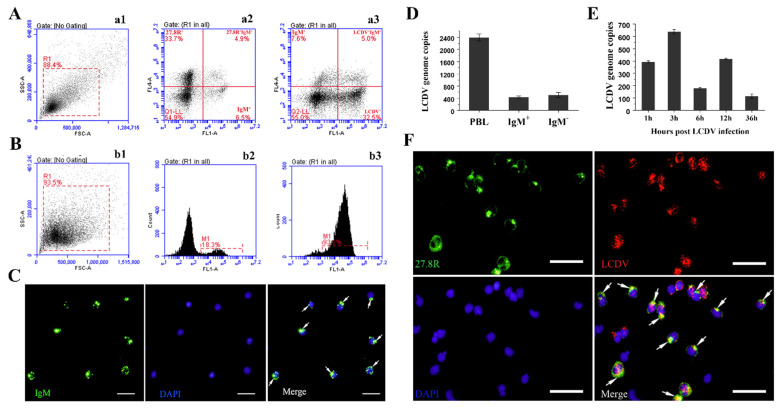
Flow cytometry analysis of 27.8R^+^/IgM^+^ and LCDV^+^/IgM^+^ cells in PBLs and LCDV detection and co-localization with the 27.8R in the sorted IgM^+^ B-lymphocytes of flounder. (**A**) Flow cytometry analysis showed the PBLs gated on a FS/SS dot plot (**a1**), the 27.8R^+^/IgM^+^ double positive PBLs (**a2**), and LCDV^+^/IgM^+^ double positive PBLs (**a3**). (**B**) FACS analysis showed the PBLs gate on a FS/SS dot plot (**b1**), IgM^+^ B-lymphocytes in PBLs before sorting (**b2**), and the sorted IgM^+^ B-lymphocytes (**b3**). M1: the ratio of IgM^+^ B-lymphocytes (**b2**), and the purity of IgM^+^ B-lymphocytes (**b3**). (**C**) The immunofluorescence staining of the sorted IgM^+^ B-lymphocytes. (**D**) The LCDV copy numbers in PBLs, the sorted IgM^+^ B-lymphocytes, and IgM^+^ B-lymphocytes-depleted (IgM^−^) PBLs at 3 hpi. Error bars represented SD (*n* = 3). (**E**) The LCDV copy numbers in sorted IgM^+^ B-lymphocytes at different time points post LCDV infection of flounder. Error bars represent SD (*n* = 3). (**F**) The co-localization of the 27.8R and LCDV in the sorted IgM^+^ B-lymphocytes. Scale bar = 10 μm.

The co-staining of 27.8R^+^ and IgM^+^ cells, LCDV^+^ and IgM^+^ cells in isolated PBLs from LCDV-infected flounder (Figure 6(a1)) were first analyzed at 3 hpi by flow cytometry. The results showed that 4.9% PBLs were stained 27.8R^+^/IgM^+^ double positive, similar to the proportion of LCDV^+^/IgM^+^ double positive PBLs (5%) (Figure 6(a2,a3)).

To further determine whether LCDV could infect IgM^+^ B-lymphocytes that expressed the 27.8R, IgM^+^ B-lymphocytes were sorted from PBLs of LCDV-infected flounder by magnetic absorption cell sorting (MACS) using anti-IgM MAbs. Flow cytometry results indicated that the purity of the sorted IgM^+^ B-lymphocytes was 93.1% (Figure 6(b1–b3)). IFA showed that the sorted B-lymphocytes presented IgM^+^ fluorescence signal on cell surface (Figure 6C). Furthermore, LCDV copies could be detected in the sorted IgM^+^ B-lymphocytes (4.11 × 10^2^ copies) and the IgM^+^ B-lymphocytes-depleted (IgM^−^) PBLs (5.43 × 10^2^ copies), which all were lower than that in the PBLs (2.41 × 10^3^ copies) (Figure 6D). At various time points post infection, LCDV copy numbers in the sorted IgM^+^ B-lymphocytes demonstrated a trend of increasing and then decreasing, exhibiting a first higher peak at 3 hpi (6.48 × 10^2^ copies) and a second lower peak at 12 hpi (4.31 × 10^2^ copies) (Figure 6E). The co-localization of the 27.8R and LCDV in the sorted IgM^+^ B-lymphocytes was further detected by IFA. The sorted B-lymphocytes were pre-incubated with AP-conjugated goat anti-mouse Ig to inhibit nonspecific combination of IgM with FITC-conjugated goat-anti-mouse Ig; the results indicated that 27.8R^+^ green fluorescence was co-localized with LCDV^+^ red fluorescence in the same IgM^+^ B-Lymphocytes, revealing that LCDV infected B-Lymphocytes that expressed the 27.8R (Figure 6F).

### 2.7. Kinetics of IgM^+^, 27.8R^+^, LCDV^+^, and LCDV^+^/IgM^+^ PBLs during Early Phase of LCDV Infection

**Figure 7:**
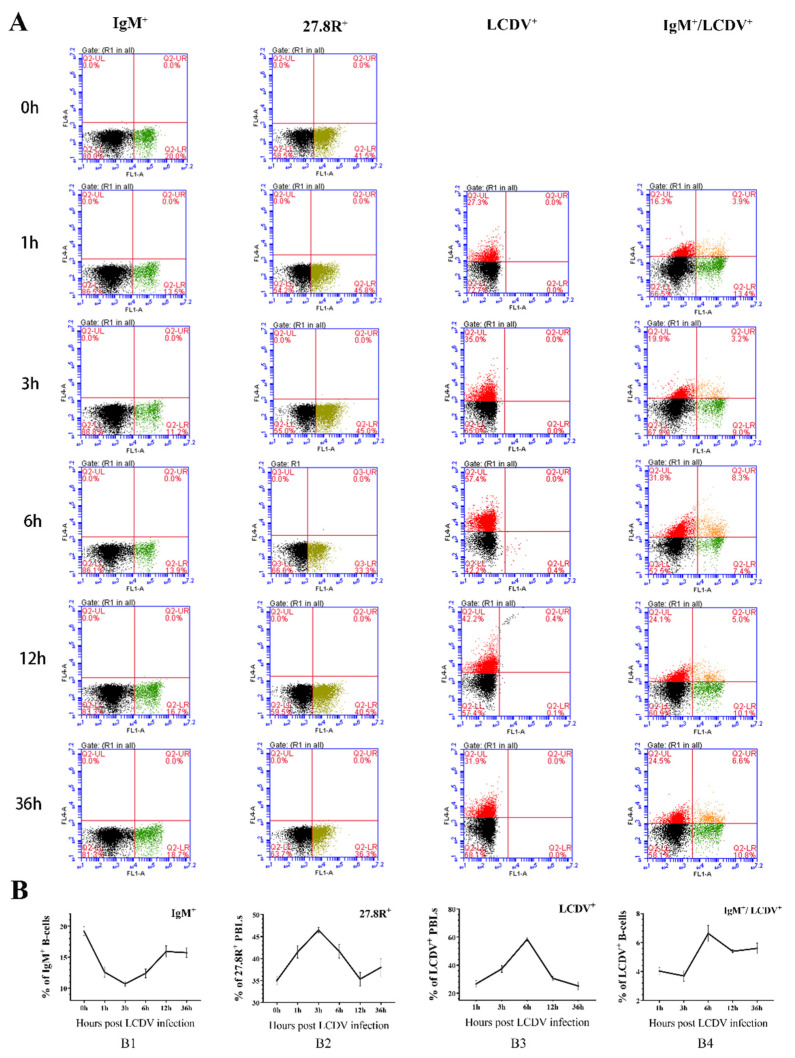
FACS analysis of the level of IgM^+^ B-lymphocytes, 27.8R^+^ PBLs, LCDV^+^ PBLs and LCDV^+^/IgM^+^ B-lymphocytes during the early phase of LCDV infection. (**A**) The proportion of various positive cells in PBLs at different time points as examples. (**B**) Time-course variations in the proportion of various positive cells in PBLs. Error bars represent SD (*n* = 3).

The PBLs were collected at 1, 3, 6, 12 and 36 h post in vivo LCDV infection of flounder, and the time-course changes of IgM^+^ B-lymphocytes, 27.8R^+^ PBLs, LCDV^+^ PBLs, and LCDV^+^/IgM^+^ B-lymphocytes were analyzed by flow cytometry (Figure 7A,B). The results indicated that the percentage of IgM^+^ B-lymphocytes in PBLs obviously declined at 1 h (12.1%) as compared to pre-infection (0 h, 19.9%), reduced to the lowest value at 3 h (10.4%), then increased at 6 h, and reached to a relatively higher level at 12 h (15.5%) and 36 h (15.0%) but still lower than before infection (Figure 7(B1)). However, the amounts of 27.8R^+^ and LCDV^+^ PBLs increased post infection, peaked at 3 h and 6 h respectively, and then decreased (Figure 7(B2,B3)). Additionally, LCDV-infected IgM^+^ B-lymphocytes in PBLs kept at about the same level from 1 h to 3 h (~4%), rose to the maximum number at 6 h (~7%) and then decreased (Figure 7(B4)), showing a similar trend to LCDV^+^ PBLs.

## 3. Discussions

In the present study, LCDV was detected in the PBLs of flounder post in vivo infection by intramuscular injection, suggesting that LCDV particles gradually entered the bloodstream from the injection site and infected the PBLs of flounder. Post in vivo and in vitro LCDV infection of PBLs, a similar change trend in LCDV copy numbers was found. The numbers increased first and then decreased, presenting a higher peak at 3 h and a second slightly lower peak at 12 h. Meanwhile, LCDV mRNA copies in in vitro infected PBLs increased and stayed at higher levels from 6 h to 24 h; also, after LCDV entry into the PBLs, virus particles at different stages of development and virions in the cytoplasm were observed under TEM. All these results revealed that LCDV could replicate in the PBLs of flounder, which could be the reason for the second peak of LCDV copies at 12 h, whereas the lower copy numbers might be due to virion release, or the disruption and apoptosis of the infected-PBLs as shown by TEM. The ability of a virus to replicate in particular cells depends on interactions between virus and cellular factors at each step of the viral cycle, from the initial entry to the ultimate release and transmission of virions. Viral receptors are the primary determinants of viral host range, tissue and cell tropism, and pathogenesis [25], and they are responsible for adsorption and penetration of the virus particle into the target cells [26]. Previously, we have identified a 27.8R from the plasma membrane proteins of FG cells and confirmed it is a functional receptor for LCDV infection [12,18], and is widely distributed in various tissues of flounder [20] and turbot [21]. LCDV is also detected in all 27.8R-positive tissues of flounder [20], more notably, LCDV, and the 27.8R is separately detected in some leukocytes but not in red blood cells of turbot [21]. However, it is not known in terms of their subsets and the role of the 27.8R in LCDV infection of these leukocytes, since the requirements for viral entry into B cells appear to be different from those for entry into epithelial cells lines as shown for the Epstein-Barr virus (EBV) [27,28]. In this research, the 27.8R was determined to express and locate on the cell surface of the leukocytes but not red blood cells of flounder, and 27.8R^+^/LCDV^+^ double positive signals were detected in the same PBLs. Blocking of the 27.8R on PBLs by anti-27.8R MAb could obviously lead to a decrease in LCDV infection, revealing that the 27.8R was also a functional receptor for LCDV infection of PBLs, which conformed to our recent finding that LCDV infection of FG cells was initiated by the interaction of the 27.8R with a 32 kDa viral attachment protein of LCDV [19]. Collectively, these results revealed that the 27.8R-expressing PBLs were permissive to LCDV infection and supported viral replication, which might serve as a vehicle for LCDV dissemination throughout the fish body and even trafficking across the blood-brain barrier, as proved in our previous study that LCDV copies increased in the brain of flounder post LCDV infection [20], resulting in the systemic infection and contributing to the pathogenesis of lymphocystis disease.

The PBLs are divided into three subpopulations, including lymphocytes, monocytes and granulocytes [29]. To clarify the specific subpopulation of PBLs permissive to LCDV infection, IEM analysis were performed in this study, it was found that the 27.8R^+^ PBLs were characterized by a large nucleus, and a thin rim of cytoplasm with small protrusions on the cell surface, which was consistent with the morphological features of the lymphocytes [30]. Additionally, LCDV^+^ fluorescence was detected in IgM^+^ and IgD^+^ PBLs, but negative in CD3^+^ PBLs, revealing that LCDV could infect IgM^+^ and IgD^+^ B-lymphocytes, but not T-lymphocytes. Nonetheless, some IgM^−^/LCDV^+^ and IgD^−^/LCDV^+^ PBLs were also found; their cell subset remains to be determined. Thus far, in rainbow trout (*Oncorhynchus mykiss*), two different subpopulations of B-lymphocytes, IgM^+^/IgD^+^/IgT^−^ and IgM^−^/IgD^−^/IgT^+^ cell, have been identified [30]; in catfish (*Ictalurus punctatus*), IgM^+^/IgD^−^, IgM^+^/IgD^+^ and IgD^+^/IgM^−^ cell have been described [31]. In flounder, however, only the IgM^+^/IgD^+^ B cell subset is identified in the PBLs by using anti-IgM and anti-IgD MAbs as probes simultaneously [32]. Therefore, the IgM^+^/LCDV^+^ cells are equivalent to IgD^+^/LCDV^+^ cells in this study, i.e., IgM^+^/IgD^+^ B lymphocytes. Although IgM, IgD and IgT have been investigated in flounder [32,33,34], no research about the B cell subset expressing surface IgT is reported because of the lack of flounder IgT antibodies. For the same reason, it remains unclear whether the IgM^−^/LCDV^+^ and IgD^−^/LCDV^+^ cell subpopulations are associated with IgT-expressing B-cells, so further research is needed in the future.

In this study, IgM^+^ B-lymphocytes were sorted from the PBLs of in vivo LCDV-infected flounder, and viral copies in the sorted cells displayed a tendency of rising in number first and then falling, with a higher peak at 3 h and a slightly lower peak at 12 h, which was consistent with the change trends of LCDV copies in PBLs. Moreover, LCDV was co-localized with the 27.8R in the same sorted IgM^+^ B-lymphocytes. All these results for the first time to our knowledge verified that IgM^+^ B-lymphocytes of teleost fish could be infected by LCDV via a receptor-mediated mechanism, and this also was the first finding for iridovirus. Notably, LCDV copies were also detected in IgM^+^ B-cells-depleted PBLs, which concurred with the presence of IgM^−^/LCDV^+^ and IgD^−^/LCDV^+^ PBLs as mentioned above. In mammals, some viruses have demonstrated a capacity to infect B-lymphocytes. EBV establishes a lifelong persistent infection in memory B-lymphocytes differentiated from latently infected B-cells in humans [35]. The virus produced in B-cells can spread to new host cells by entry and replication in new epithelial cells [36], while EBV lytic reactivation typically occurs in B-cells expressing either IgM or class-switched IgG heavy chains [37]. Murine gamma herpesvirus 68 (MHV68) has tropism for B-lymphocytes [38] and persists in memory B cells [39]. To persist in an infected individual lifelong, these herpesviruses have developed multiple strategies to modulate innate and adaptive immunity [37]. Recently, primary human B-cells at different differentiation and maturation stages are found to exhibit distinct susceptibilities to vaccinia virus binding and infection, and the infections are abortive and productive in ex vivo and activated B cells, respectively [40]. Moreover, human B cells are susceptible to direct infection by dengue virus (DENV) via CD300a and support viral replication albeit low titers of infectious virions were released [41]. Interestingly, LCDV can be detected in fish showing no clinical signs of lymphocystis disease and persists in the epidermal tissues for more than 2 months at low temperatures [42]. Combining our results in this study, we hypothesize that B-cells may play a role in persistent infection of LCDV in teleost fish, but further researches are required to clarify this. In teleosts, the immediate effects of viral encounter on B-cells have been scarcely addressed. Viral hemorrhagic septicemia virus (VHSV) has been found to infect rainbow trout spleen IgM^+^ B cells, although the infection is not productive and just viral transcription is detected, which points to VHSV-induced IgM^+^ B-cell activation towards an antigen-presenting profile [43]. B-cells are a main type of antigen-presenting cells in addition to producing antibodies. References reveal that the primary IgM^+^ B cells of teleosts have potent phagocytic ability [44], and all teleost species analyzed thus far have phagocytic B cells, suggesting that this is a general feature of teleost B cells [44,45]. Previously, we also have found that B-lymphocytes of flounder are capable of phagocytosis [46]. In this research, incubation of UV-inactivated LCDV with the PBLs, which gave significantly lower viral copy numbers than incubation of productive LCDV, eliminated the influence of the antigen phagocytosis by PBLs. In other words, the 27.8R-expressing B-cells in flounder served as target cells of LCDV infection but not just as antigen-presenting cells. Of course, the antigen-presenting function of B-cells in LCDV-infected flounder needs to be further investigated.

In the present study, the amounts of the 27.8R^+^ PBLs increased post in vivo infection and peaked at 3 h, indicating that LCDV infection upregulated the expression of 27.8R on PBLs, as previously described in FG cells and in various tissues of flounder and turbot [18,20,21]. Moreover, LCDV^+^ PBLs and LCDV-infected IgM^+^ B-cells had a proportional increase and arrived at the peak value at 6 h, and a higher rate in the percentages was exhibited for 27.8R^+^ and LCDV^+^ PBLs than LCDV-infected IgM^+^ B-cells, further verifying that other PBLs expressing the 27.8R could be infected except IgM^+^ B-cells, which agreed with the existence of IgM^−^/LCDV^+^ PBLs and LCDV detection in IgM^+^ B-cells-depleted PBLs aforementioned. Although just a small population of IgM^+^ B-cells in PBLs were infected by LCDV (from 4% at 1 h to 7% at 6 h), the amount of IgM^+^ B-cells clearly declined at 1 h (12.1%) as compared to pre-infection (19.9%), reduced to the lowest value at 3 h (10.4%), and then increased but was still lower than pre-infection at 12 h and 36 h (~15%), revealing that LCDV infection facilitated damage to B-cells during the early stage, as proved by TEM results that showed karyopyknosis of the PBLs, cell disruption and apoptosis. These results are considered in two ways. Firstly, the effects of virus infection on B lymphocyte function and, secondly, the effects of residence in B lymphocytes which serve as immune cells on the biological properties of viruses. B cells are a highly heterogeneous population of cells at different stages of maturation and differentiation, each with unique functional properties and cell surface phenotypes; hence, the mechanism by which direct infection by LCDV alters B cell responses needs to be further clarified. As for whether apoptosis is induced in the LCDV-infected cells, references indicate that LCDV induces apoptotic cell death in vitro in FG cells, as demonstrated by cell nucleus chromatin condensation, chromosomal DNA fragmentation and caspase activation [47]. In our previous research, transcript analysis of flounder gills after LCDV infection shows the differential expression of apoptosis-related genes (e.g., TNF ligand superfamily member 13B and TNF receptor-1) and pathways (proteasome, p53 signaling pathway, and TNF signaling pathway) [17]. Upon in vivo infection, LCDV can produce a cytoplasmic TNF receptor (TNFR)-like protein which may interact with one or more apoptosis or proliferation signaling molecules, and thereby inhibit the apoptosis cascade downstream of the TNFR superfamily [48]. In addition, LCDV-infected cells are found to be inhibited in apoptotic death and cell division before enlargement in the early stage of lymphocystis cell formation [49]. LCDV isolated in China is reported to contain a putative homolog of cellular G protein-coupled receptors, which may inhibit apoptosis, as the viral G protein-coupled receptors can help the virus escape from host immune surveillance and contribute to viral pathogenesis [50]. A TNF receptor analog has been identified in LCDV [4], while VDAC can be oligomerized by TNF-α. Whether TNF-α modulates VDAC behavior in LCDV^+^ cells, and the mechanisms for the induction of apoptosis of LCDV^+^ cells in vivo and in vitro are the subject of further studies, which would be of great biological significance if LCDV hijacks the apoptotic pathway.

We have previously confirmed that 27.8R is the VDAC2 and RACK1 of flounder [22]. VDAC is originally characterized as abundant proteins in the outer mitochondrial membrane of cells, which might be why we did not observe the 27.8R in erythrocytes which are devoid of mitochondria, while it was observed in PBLs in this study. However, a relatively recent understanding is that a specific isoform of VDAC (i.e., VDAC1) has been also found on the plasma membrane of human B-lymphocytes [51], which is consistent with our result that the 27.8R as an isoform of VDAC was observed abundantly in the plasma membrane of PBLs. VDAC2 is one of the isoforms of VDACs, along with VDAC1 and VDAC3 in mammals. In our previous studies, it is found that both VDAC1 and VADC2 in the plasma membrane proteins of FG cells can react with 27.8R MAbs, but VDAC1 cannot react with LCDV and the 32 kDa viral attachment protein that specifically interacts with 27.8R. This implies that VDAC1 is not involved in LCDV infection, although VDAC1 may have an antigen epitope similar to that of VDAC2 [22]; moreover, caveolin-1 on FG cell membrane is co-localized with LCDV, VDAC2, and RACK1, and LCDV enters into FG cells via caveolae-mediated endocytosis facilitated by VDAC2 and RACK1 receptors [15]. All these results verify that VDAC2 and RACK1 are located on the plasma membrane; however, whether LCDV enters into B-lymphocytes via the same mechanism as into FG cells remains to be investigated. As to why anti-27.8R MAb did not detect erythrocyte VDAC in its plasma membrane and mitochondrial VDAC in the PBLs of flounder in this study, further research is needed to clarify this. Furthermore, it has been reported that Japanese encephalitis virus (JEV) infection causes a redistribution of VDAC and suggests this may be a part of the JEV replication strategy in insect cells [52]. We have found the mRNA expressions of VDAC2 and RACK1 in FG cells are upregulated by LCDV infection [22]; however, whether VDAC2 and RACK1 also are relocalized in response to LCDV infection in B-lymphocytes and other permissive cells of flounder is worthy of more studies.

Taken together, the current study investigated the role of the PBLs in LCDV infection. We found that the PBLs that expressed the 27.8R on the cell membrane were permissive to LCDV infection and supported viral replication, and the 27.8R functioned as a receptor for LCDV infection of PBLs. Further evidence indicated that the 27.8R-expressing IgM^+^ B lymphocytes, but not T lymphocytes, acted as target cells of LCDV infection, whereas the cell subsets of some IgM^−^/LCDV^+^ and IgD^−^/LCDV^+^ leukocytes remained to be determined. Previously, fibroblasts, hepatocytes and macrophages of the mononuclear phagocyte system have been suggested to be target cells for LCDV replication [53,54]. Thus, this novel finding that IgM^+^/IgD^+^ B-lymphocytes were involved in LCDV infection via cellular receptor-mediated mechanism would extend the cellular tropism of LCDV and provide a better understanding of the roles of teleost B-cells in LCDV transmission and viral pathogenesis.

## 4. Materials and Methods

### 4.1. Ethics Statement

This study was carried out in strict accordance with the recommendations of the Guidelines for the Use of Experimental Animals of Ocean University of China. The protocol was approved by the Committee on the Ethics of Animal Experiments of Ocean University of China (permission number: 20190101).

### 4.2. Fish, Virus and Antibodies

Healthy flounders (*P*. *olivaceus*) weighing about 600 g were obtained from a local fish farm in Qingdao of Shandong province, China. Fish were confirmed as LCDV-free via PCR [55], and kept in rearing tanks supplied with running aerated seawater at 20 ± 1 °C and fed daily with dry food pellets before use.

Virus strain LCDV-HD (GenBank accession number: DQ279090) was purified in previous studies and stored at −80 °C [19,56]. The final concentration of virus was adjusted to 1 mg in 1 mL sterile phosphate buffered saline (PBS, pH 7.4), and the 50% tissue culture infective dose (TCID_50_) was determined using Reed–Muench method [57].

Mouse anti-27.8R MAb 2G11 and rabbit anti-27.8R polyclonal antibody [14], mouse anti-LCDV MAb [58], rabbit anti-LCDV polyclonal antibody [12], mouse anti-WSSV MAb 1D5 [59], mouse anti-flounder IgM MAb 2D8 [33], mouse anti-flounder IgD MAb [32], and mouse anti-flounder CD3ε polyclonal antibody recognizing T-lymphocytes [60], were previously produced by our laboratory and used in this experiment.

### 4.3. In Vivo LCDV Infection and Blood Cell Sampling

The fish were divided into two groups (with three replicates in each group), and injected intramuscularly with purified LCDV (300 μL per fish) or the same volume of PBS as negative control. Six fish in each group were randomly sampled at 1, 3, 6, 12 and 36 hpi, and the peripheral blood was drawn from the caudal vein of flounder which were sacrificed via MS-222 (Sigma, St. Louis, MO, USA) overdose, and diluted 1:1 in 65% RPMI-1640 (Gibco, Darmstadt, Germany) containing 20 IU/mL heparin (Sangon Biotech, Shanghai, China). The peripheral blood from LCDV-infected fish and the PBS-control group was used for isolation of PBLs by Percoll density gradient centrifugation as previously described [33]. Briefly, the cell suspensions were laid over a discontinuous Percoll gradient at densities of 1.020 and 1.070 g/cm^3^ and centrifuged at 840× *g* for 30 min at 4 °C. The lymphocytes fraction was collected and washed twice with PBS containing 5% (*v*/*v*) newborn calf serum (Hyclone, Logan, UT, USA) at 640× *g* for 10 min, and then resuspended in PBS for next use.

### 4.4. Indirect Immunofluorescence Assay

For LCDV detection by IFA, the isolated PBLs from LCDV-infected flounder were adjusted to 10^6^ in 1 mL PBS and settled on glass slides for 2 h subsiding, followed by fixing with 4% paraformaldehyde for 20 min. The slides were blocked by 3% bovine serum albumin (BSA) in PBS for 1 h at 37 °C, and washed three times with PBS containing 0.05% Tween-20 (PBST). Subsequently, cells were incubated with mouse anti-LCDV MAb (1:1000) as primary antibody for 1 h at 37 °C, and with anti-WSSV MAb 1D5 (1:1000) as negative control. After three washes, the PBLs were incubated with fluorescein isothiocyanate FITC-conjugated goat-anti-mouse Ig (1:256, Sigma, USA) for 1 h at 37 °C in the dark. DAPI (Roche, Basel, Switzerland) staining was used to visualize the cell nuclei.

### 4.5. Flow Cytometry Analysis

The isolated PBLs from LCDV-infected flounder were incubated with mouse anti-LCDV MAb (1:1000) for 1 h at room temperature with gentle shaking. Subsequently, cells were rinsed thrice with PBS by centrifugation at 640× *g* for 5 min, followed by incubation with goat anti mouse Ig-FITC (1:256, Sigma, USA). After washing three times with PBS, the final PBLs suspensions were analyzed with a flow cytometry (Beckman Counter, Brea, CA, USA).

### 4.6. Real-Time Quantitative PCR

To determine the changes of LCDV copy numbers in PBLs, the viral genomic DNA (gDNA) in PBLs from LCDV-infected flounder at 1, 3, 6, 12, and 36 hpi was extracted with a MiniBEST Viral DNA Extraction Kit Ver.5.0 (TaKaRa, Kusatsu, Japan) according to the manufacturer’s instruction, and LCDV genome copies in PBLs of flounder were detected by qPCR. The specific primers P1/P2 were designed as described before according to the sequence of the LCDV ORF038 gene: 5′-TCT TGT TCA GCA TTT ACT TCT CGGC-3′, 5′ TCT TCT CCTTA GAT GAT TTC CC-3′ [19]. The qPCR reaction system contained 10 μL SYBR qPCR Master Mix (Roche, Switzerland), 2 μL primer P1/P2, and 50 ng template DNA, adding sterile distilled water to a final volume of 20 μL. The reaction procedures were as follows: 10 min at 95 °C, then 45 cycles of 10 s at 95 °C, 10 s at 55 °C and 20 s at 72 °C. The LCDV copy numbers were calculated according to the Ct values via the standard curve generated before [19].

### 4.7. In Vitro LCDV Infection of PBLs and Detection of Viral Replication

The isolated PBLs from healthy flounder in the control group were suspended in Leibovitz’s L-15 medium (Gibco, Darmstadt, Germany) supplemented with 2% fetal bovine serum (FBS), 100 IU/mL penicillin and 100 μg/mL streptomycin (Gibco, Darmstadt, Germany), and cultured in 6-well plates. For LCDV infection assay, plates were inoculated with LCDV at 4 TCID_50_ for 1 h [14]. After virus adsorption, the unattached viral particles were removed by washing with L-15 medium without FBS, and maintenance medium was added. To confirm whether LCDV could proliferate in PBLs, the PBLs were collected at 1, 3, 6, 12, 24 and 36 hpi and divided into two groups; one was used for LCDV gDNA extraction as described above, another was for mRNA extraction with TRIzol Reagent (Thermo Fisher Scientific, Waltham, MA, USA) and then reverse-transcribed into cDNA using PrimeScript RT Master Mix (TaKaRa, Kusatsu, Japan). The LCDV gDNA and reverse-transcribed cDNA were detected by qPCR as -mentioned above. Considering the fact that B-lymphocytes of flounder are capable of phagocytosis [46], UV-inactivated LCDV particles (UV-LCDV), which were prepared under UV irradiation for 30 min [41,61], were used as control to eliminate the influence of the antigen phagocytosis on LCDV copy numbers.

Furthermore, the infected PBLs were sampled at 1, 3, 6, 12 and 36 hpi after washing thrice in PBS, and fixed with 2.5% glutaraldehyde in PBS for 2 h at 4 °C and post-fixed with 1% osmium tetroxide. Subsequently, ultrathin sections were routinely processed and observed under transmission electron microscope.

### 4.8. Co-Staining of LCDV with the 27.8R on PBLs

The slides of whole blood cells from LCDV-infected flounder were first prepared and used for detection of the 27.8R by IFA by using mouse anti-27.8R MAb (1:1000) and goat anti-mouse Ig-FITC (1:256) as primary and secondary antibodies, and the cells were counterstained by 1 μg/mL Evans blue dye (EBD, Fluka). To further determine whether LCDV co-localized with the 27.8R on the same PBLs, the slides of the isolated PBLs at 3 hpi were prepared as described above, then co-immunofluorescence staining of LCDV and the 27.8R was performed on the same slide. Rabbit anti-LCDV polyclonal antibody (1:500) paired with mouse anti-27.8R MAb (1:1000) served as primary antibodies, and rabbit pre-immune serum paired mouse pre-immune serum served as negative controls. After three washes, FITC-conjugated goat-anti-mouse Ig (Sigma, St. Louis, MO, USA) and Cy3-labeled goat-anti-rabbit (Sigma, St. Louis, MO, USA) at a dilution ratio of 1:256 in PBS were added as secondary antibodies. Following three washes, DAPI (Roche, Basel, Switzerland) staining was performed to visualize cell nuclei. Slides were rinsed again, and then mounted with 90% glycerin and observed under a fluorescence microscope.

Additionally, the PBLs from LCDV-infected flounder were incubated with mouse anti-27.8R MAb 2G11 (1:2000) paired with anti-LCDV polyclonal antibody (1:500) for 1 h, followed by incubation with goat anti mouse Ig-FITC (1:256, Sigma, USA) paired with Alex 647 goat-anti-rabbit Ig (1:256, Thermo Fisher, Waltham, Massachusetts, USA) for 1 h in the dark. Rabbit pre-immune serum paired with mouse pre-immune serum served as negative control. After washing three times with PBS, the final PBLs suspensions were analyzed with a flow cytometry as described above.

### 4.9. Immunogold Electron Microscopy

For location of the 27.8R on PBLs and morphologic observation of the 27.8R^+^ PBLs in healthy flounder, the PBLs in the control group were fixed with 2.5% glutaraldehyde in PBS at 4 °C for 2 h. After washing three times with PBS, the cells were post-fixed with 1% osmium tetroxide, dehydrated with a graded ethanol series and embedded in Epon 812. Ultrathin sections were prepared and picked up on nickel grids, washed with distilled water for 5 min, and incubated with 1% H_2_O_2_ for 10 min. After three washes in double distilled water and then in PBS, the sections were treated with 3% BSA in PBS for 1 h at 37 °C. Following three washes in PBST, they were incubated with anti-27.8R MAb 2G11 (1: 1000) for 1 h at 37 °C, and anti-WSSV MAb 1D5 (1: 1000) was used as negative control. The sections were rinsed in PBST again, and incubated with 10 nm colloid gold conjugated goat-anti-mouse Ig (Sigma, USA) for 1 h at 37 °C. After three washes, the sections were stained with uranyl acetate and then lead citrate for 30 min, respectively. Finally, the sections were subjected to three washes in PBST and double distilled water, and observed under a transmission electron microscope.

### 4.10. In Vitro Competition Assay between LCDV and Anti-27.8R MAb

To further identify whether the 27.8R was a functional receptor involved in LCDV infection of PBLs, an antibody-blocking assay using anti-27.8R MAb and isotype control was carried out in PBLs in vitro. The PBLs from healthy flounder were incubated with mouse anti-27.8R MAb at concentrations of 0.12 μg/mL, 1.2 μg/mL, and 12 μg/mL for 2 h, respectively, and the same volume of mouse anti-WSSV MAb 1D5 was used as controls. After three washes with PBS, the PBLs were inoculated with 100 μL LCDV at 4 TCID_50_/mL. Following three washes again, the cells were collected at 24 h for DNA extraction using MiniBEST Viral DNA Extraction Kit Ver.5.0 (TaKaRa, Japan) for detection of LCDV copy numbers by qPCR as above.

### 4.11. Multicolor Fluorescence Microscopy

To determine the leukocyte subsets of LCDV-positive PBLs, the slides of PBLs from LCDV-infected flounder at 3 hpi were prepared as above, rabbit anti-LCDV polyclonal antibody (1:500) was paired with mouse anti-IgM MAb (1:2000), anti-IgD MAb (1:1500), and anti-CD3ε polyclonal antibody (1:400) as primary antibodies, respectively, and rabbit non-immune paired with mouse non-immune serum were used as negative control. After the cells were washed three times with PBST, FITC-conjugated goat-anti-mouse Ig (Sigma, USA) and Cy3-labeled goat-anti-rabbit Ig (Sigma, USA) at a dilution ratio of 1: 256 in PBS were added as secondary antibodies. Following incubation for 1 h at 37 °C, the cells were rinsed again and DAPI (Roche, Switzerland) staining was carried out to visualize cell nuclei. Slides were rinsed again, and then mounted with 90% glycerin and observed under a fluorescence microscope.

### 4.12. Magnetic Absorption Cell Sorting

To further confirm that the peripheral blood B-lymphocytes expressed the 27.8R and acted as a target for LCDV infection, peripheral blood B-lymphocytes were sorted from the PBLs of LCDV-infected flounder by magnetic absorption cell sorting (MACS) with the following procedures. The PBLs were first incubated with anti-IgM Mab 2D8 (1:2000) at 37 °C for 1.5 h and rinsed thrice with PBS for 5 min each, followed by incubation with 100 μL anti-mouse IgG microbeads (Miltenyi Biotec, Bergisch Gladbach, Germany) at 4 °C for 15 min, and then resuspended with PBS after triple rinsing. The PBLs suspension was added to the LS sorting column (Miltenyi Biotec) which was installed at MACS manual separator (Miltenyi Biotec). The PBLs which were not labeled by immunomagnetic beads would flow out first and were collected with a clean centrifuge tube. After that, the sorting column was removed from the manual separator and rinsed quickly with PBS to collect the immunomagnetic bead labeled cells, i.e., IgM^+^ B-lymphocytes. The purity of the sorted peripheral blood B-lymphocytes was detected by flow cytometry and IFA as described above. Furthermore, IgM^+^ B-lymphocytes and the depleted PBLs (IgM^−^) were collected and adjusted to 1 × 10^7^ cells/mL, and then DNA extraction using TIANamp Marine Animals DNA Kit (Qiagen, Hilden, Germany) was carried out to detect LCDV copy numbers by qPCR. The smear slides of IgM^+^ B-lymphocytes were prepared, and immunofluorescence co-staining of LCDV with the 27.8R was performed as aforementioned.

### 4.13. Detection of IgM^+^, 27.8R^+^, LCDV^+^, and LCDV^+^/IgM^+^ PBLs at Different Time Points

Before LCDV infection of flounder, the PBLs were isolated and incubated with mouse anti-27.8R MAb 2G11 (1:2000), or anti-IgM MAbs 2D8 (1:2000) paired with rabbit anti-27.8R polyclonal antibody for 1 h, followed by incubation with goat anti-mouse Ig-FITC, or goat anti-mouse Ig-FITC paired with Alex 647 goat anti-rabbit Ig for 1 h in the dark, and analyzed with a flow cytometry as mentioned above. Post LCDV in vivo infection, the PBLs were collected at 1, 3, 6, 12, and 36 hpi, and fixed with 4% paraformaldehyde at 22 °C for 20 min. Cells were separately incubated with mouse anti-27.8R MAbs, rabbit anti-LCDV polyclonal antibody, mouse anti-IgM MAb 2D8, and rabbit anti-LCDV polyclonal antibody paired with mouse anti-IgM MAb 2D8 as primary antibodies, and then incubated with FITC-conjugated goat-anti-mouse Ig or Alex 647 goat-anti-rabbit Ig as secondary antibodies. Finally, the percentage of 27.8R^+^ PBLs, LCDV^+^ PBLs, IgM^+^ B-lymphocytes, and LCDV-infected IgM^+^ B-lymphocytes in PBLs were detected by flow cytometry.

### 4.14. Statistical Analysis

All data was expressed as mean ± standard deviation. The statistical analysis was performed using GraphPad Prism version 7.00 Software (GraphPad Software, La Jolla California, USA) and one-way ANOVO. Differences were considered statistically significant at *p* < 0.05.

## Data Availability

The data presented in this study are available upon request from the corresponding author.
